# Initiating factors for the onset of OA: A systematic review of animal bone and cartilage pathology in OA

**DOI:** 10.1002/jor.24605

**Published:** 2020-02-13

**Authors:** Michelle E. Casper‐Taylor, Andrew J. Barr, Sophie Williams, Ruth K. Wilcox, Philip G. Conaghan

**Affiliations:** ^1^ School of Mechanical Engineering, Institute of Medical and Biological Engineering University of Leeds Leeds UK; ^2^ NIHR Leeds Biomedical Research Centre, Leeds Institute of Rheumatic and Musculoskeletal Medicine University of Leeds Leeds UK

**Keywords:** bone, cartilage, knee, osteoarthritis

## Abstract

There is controversy over whether bone or cartilage is primarily involved in osteoarthritis (OA) pathogenesis; this is important for targeting early interventions. We explored evidence from animal models of knee OA by preforming a systematic review of PubMed, Scopus, and Web of Science for original articles reporting subchondral bone and cartilage pathology in animal models with epiphyseal closure. Extracted data included: method of induction; animal model; cartilage and bone assessment and method; meniscal assessment; skeletal maturity; controls; and time points assessed. Quality scoring was performed. The best evidence was synthesized from high‐quality skeletally mature models, without direct trauma to tissues of interest and with multiple time points. Altogether, 2849 abstracts were reviewed. Forty‐seven papers were included reporting eight different methods of inducing OA, six different species, six different methods of assessing cartilage, five different bone structural parameters, and four assessed meniscus as a potential initiator. Overall, the simultaneous onset of OA in cartilage and bone was reported in 82% of datasets, 16% reported bone onset, and 2% reported cartilage onset. No dataset containing meniscal data reported meniscal onset. However, using the best evidence synthesis (n = 8), five reported simultaneous onset when OA was induced, while three reported bone onset when OA occurred spontaneously; none reported cartilage onset. In summary, there is a paucity of well‐designed studies in this area which makes the conclusions drawn conjectures rather than proven certainties. However, within the limitation of data quality, this review suggests that in animal models, the structural onset of knee OA occurs either in bone prior to cartilage pathology or simultaneously.

**Abbreviations**
AB: Andrew Barr
ACLT: anterior cruciate ligament transection
BMD: bone mineral density
BV/TV: bone volume fraction
MC: Michelle Casper‐Taylor
OA: osteoarthritis

## 1 INTRODUCTION

Osteoarthritis (OA) is typically diagnosed and treated when pathology becomes detectable by conventional radiography, but the OA process begins long before this as evidenced by magnetic resonance imaging (MRI)‐detectable structural pathology.^1^ Therapies targeting tissue pathology at an earlier stage of disease (eg, after a major injury or in individuals at risk) might permit disease modification.

The concept of early OA in humans is imprecisely defined, and limited data exist on the timing and sequence of events that occur at the onset of human knee OA. OA is a syndrome of deterioration of synovial joints, characterized by multitissue pathology involving hyaline cartilage, subchondral bone, the calcified cartilage layer, the synovium and capsule, articular ligaments (in knees), and the menisci. However, there remains considerable controversy over which tissue is key to initiate the OA process.^2^ In humans, subchondral bone abnormalities,^3^ cartilage lesions,^4^ and meniscal damage^3^, ^5^ are all present in the knees of adults prior to radiographic OA. Nonetheless, the presence of these tissue lesions is associated with subsequent structural deterioration^6^, ^7^, ^8^ and incident radiographic knee OA.^9^

In light of ethical considerations surrounding human tissue sampling, animal models of OA are essential to advance the understanding of the temporal connection between cartilage pathology, subchondral bone structure, and menisci in the genesis and propagation of OA. Studying animal models is complex because in addition to the numerous different types of animals, there are numerous models of OA, some spontaneous and some induced. Often models are used to explore different mechanistic aspects of OA.

We aimed to perform a systematic review to evaluate animal studies of knee OA which reported data concerning subchondral bone structure along with cartilage and meniscal pathology at the onset of OA to assess the sequence of events occurring at the onset of knee OA.

## 2 METHODS

### 2.1 Search strategy and selection of studies

Three databases (PubMed, Web of Science, and Scopus) were searched up to April 2018. The keywords used were: bone, cartilage, OA, osteoarthrosis, arthrosis, arthritis, animal, and animals. Filters excluding articles not written in English, studying humans and review articles were applied and filters including animals and full articles were applied and were available for each database (Supporting Information A). Meniscus and synovium were not included in the search terms because earlier exploratory searches highlighted the relative paucity of studies including bone, cartilage, and meniscal analysis. Had meniscus been included in the search terms, only four studies would have been eligible for data extraction. Additionally, had synovium been included, no studies would have been eligible for data extraction. Therefore, only bone and cartilage search terms were used and we evaluated meniscal data when available as a part of the data extracted from the included studies.

Initially, titles and abstracts were screened and excluded if they: (a) used animals from the Muroidea superfamily; (b) used nonstandard animals (eg, python, sea lion, etc); (c) did not specify animal model; (d) did not present bone structure and cartilage degradation data; (e) conducted a surgical repair; (f) were conducted on joints other than the knee or stifle; (g) were conducting a study to see the effect of a given intervention; (h) induced OA through chemical injection of chondrotoxic or other proinflammatory substances; (i) did not induce OA; or (j) were a case study (n = 1). Additionally, articles were manually filtered for nonanimal, review articles, book chapters, and editorials. Full articles were then retrieved and the references screened for potential studies missed by the literature search.

We structured this review to exclude animal models in the Muroidea superfamily (mice and rats). Although mice and rats are commonly used for biomaterials testing at early stages of OA, their thin cartilage layer (3‐5 cells thick) and the small nature of their joints^10^, ^11^, ^12^, ^13^, ^14^ make it difficult to discern changes in bone structural parameters that are comparable to the human OA disease. Additionally, their lack of growth plate fusion makes it difficult to distinguish whether changes in bone structural parameters result solely from OA or from the influence of persistent growth plate patency.^15^, ^16^, ^17^ Moreover, while these rodent models are useful for basic research because they are small, inexpensive, easy to manipulate on a genetic level, feature short reproduction cycles and lifespans, and produce meaningful and relevant data,^18^ several articles and reviews highlight the poor correlation of rodent models with a variety of different human conditions by demonstrating failure to successfully translate rodent‐based findings into successful clinical practice.^15^, ^16^, ^17^ To remain focused and avoid ambiguity, we have decided to exclude these animal models. Guinea pigs, in contrast, were included in this review because OA in guinea pigs more closely resembles human knee OA pathogenesis ^19^ and because guinea pig growth plates fully fuse.

### 2.2 Data extraction

Data extracted included: method of OA induction; animal species; methodology of measurements; assessment of cartilage degeneration; assessment of bone structure; assessment of meniscus; skeletal maturity; experimental control; number of relevant time points; and origin of OA pathology. If a study involved more than one method of inducing OA, datasets were extracted separately for each method. We only recorded changes from control (or baseline) from the cartilage degradation and bone structure data, which was presented in the extracted datasets. Data were extracted and tabulated by one reviewer (Michelle Casper‐Taylor [MC]) and then a subset of key variables was validated by an additional reviewer (Andrew Barr [AB]).

### 2.3 Data analysis

Histological and imaging structural bone and cartilage data were assessed to determine which tissue degenerated first or whether they degenerated simultaneously. Cartilage and bone involvement were defined as pathology differing from baseline or control. Datasets were stratified according to whether subchondral bone and cartilage structural pathology occurred concurrently (simultaneous onset) or in series with bone first (bone onset) or cartilage first (cartilage onset). A meta‐analysis was precluded because of the heterogeneity of the datasets which were included.

### 2.4 Quality analysis

The quality of each dataset was determined using a combination of several quality scoring systems that were modified to assess dataset quality and potential bias in study design and reporting of cartilage degradation and subchondral bone structural data.^20^, ^21^, ^22^, ^23^, ^24^ The checklist was comprised of 11 criteria (Supporting Information B). Each criterion received a score of 1 if it was reported satisfactorily and received a 0 if it was not. The maximum score was 11. Scores were tabulated by two reviewers independently (MC and AB) and then compared. Discrepancies in scoring were discussed until consensus was reached. Datasets were then rated as having high (9‐11), medium (6‐8), or low (0‐5) quality based on their final quality score.

Best evidence synthesis was derived from selecting high‐quality studies, using models without direct trauma to tissues of interest (ie, meniscectomy, groove model, subchondral fracture, and osteochondral fragment), with skeletally mature animals and tissue data at multiple time points.

### 2.5 Statistical analysis

Statistical analysis was not preformed since there were not sufficient best evidence synthesis datasets. The analysis would have been underpowered, and, thus, would have provided no meaningful insight.

## 3 RESULTS

### 3.1 Study selection and characterization

**Figure 1:**
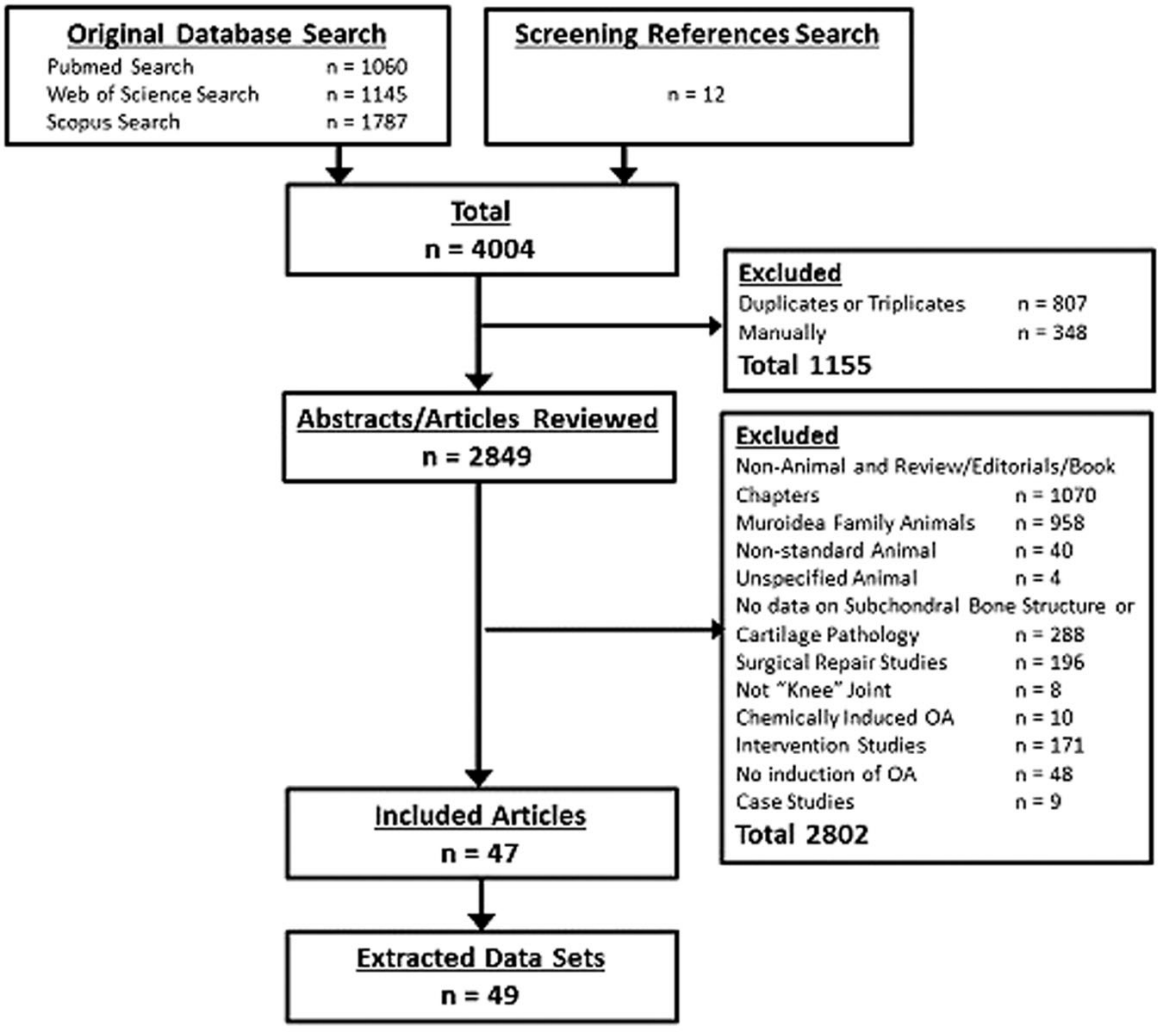
PRISMA flowchart detailing study selection and exclusion criteria

The PRISMA flowchart describing study selection is presented in Figure 1. The search returned 4004 articles. Replicate removal resulted in 2849 articles being included. Inclusion/exclusion criteria were applied and they were manually filtered to exclude nonanimal, review, and editorial articles, as well as book chapters. After reviewing the reference sections of the resulting articles, an additional 12 articles were added and screened. In total, 47 articles were included (Supporting Information C) and from these, we were able to extract 49 datasets.

### 3.2 Data extraction from selected studies

A summary of key characteristics of these datasets is presented in Table 1 and the extracted data from each dataset are presented in Table S1 and Supporting Information C. The majority of datasets used were either the anterior cruciate ligament transection (ACLT) model (16/49) or spontaneous models of OA (19/49). The most common animals used were guinea pig (16/49), dog (14/49), and rabbit (12/49). While most datasets had animals that had reached skeletal maturity (40/49), there were several datasets that studied animals that reached skeletal maturity during the duration of the study (6/46), and two in which the animals never reached skeletal maturity. One dataset did not state whether or not the animals were skeletally mature.

**Table 1:** Summary of study characteristics

Study characteristic	Subgroups	Number of studies
Species	Guinea pig	16
	Dog	14
	Rabbit	12
	Horse	5
	Cat	1
	Sheep	1
OA model	Spontaneous	19
	ACLT	16
	Groove model	4
	Meniscectomy	4
	Overloading	3
	Subchondral fracture	1
	Medial Release	1
	Osteochondral fragment	1
Relative time points	Single	19
	Multiple	30
Skeletal maturity	Skeletally mature	40
	Skeletally mature by end of study	6
	Not skeletally mature	2
	Unknown	1
Cartilage degradation^a^	Mankin or modified Mankin	24
	MRI	11
	Histologic characterization	6
	Histologic scoring	3
	OARSI	6
	Macroscopic scoring	1
Bone structural parameters^a^	BMD	18
	BV/BT	22
	Subchondral plate thickness	27
	Defect identification	18
	Stiffness	6

Abbreviations: ACLT, anterior cruciate ligament transection; BMD, bone mineral density; BV/BT, bone volume fraction; MRI, magnetic resonance imaging; OA, osteoarthritis; OARSI, Osteoarthritis Research Society International.

^a^ Datasets may be counted in more than one subgrouping.

There were six different methods used for assessing cartilage degeneration: Mankin/modified Mankin (24/49); MRI (11/49); histological characterization (6/49); OARSI scoring (6/49); histological scoring (other than Mankin or OARSI, 3/49); and macroscopic descriptive characterization (1/49).

Five different structural parameters of bone were reported: subchondral plate thickness (27/49); bone volume fraction (BV/BT, 22/49); bone mineral density (BMD, 18/49); defects such as bone marrow lesions/oedema, subchondral bone sclerosis, and subchondral bone cysts (18/49); and stiffness (6/49).

### 3.3 Quality analysis

**Figure 2:**
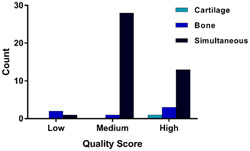
Summary of origin of osteoarthritis according to quality score [Color figure can be viewed at wileyonlinelibrary.com]

There were 17 datasets of high quality, 29 datasets were of medium quality, and 3 were of low quality. Individual scores are presented in Supporting Information D. The mean quality score was 7.6 (range, 5‐11) which was considered to be “medium” quality. Half of the datasets demonstrating bone onset were of high quality (3/6), one was of medium quality, and two were of low quality. The one dataset demonstrating cartilage onset was a high‐quality study. Most datasets demonstrating simultaneous onset were of medium quality 28/42. There was one of low quality and 13/42 was of high quality. This data are summarized in Figure 2. To the high‐quality datasets, additional criteria were applied, as outlined in Section 2. Eight of these datasets met the additional criteria and were used for best evidence synthesis analysis (8/17).

### 3.4 Data analysis of the onset of OA pathology: All studies

Structural pathology was most commonly described occurring simultaneously in subchondral bone and cartilage (42/49 datasets). Subchondral bone onset was reported in 6/49, while only one set reported cartilage onset. Using our best evidence synthesis group (n = 8), no dataset reported cartilage onset, three datasets reported bone onset Supporting Information C #2, #33, #36 and five reported simultaneous onset Supporting Information C #4, #5, #9, #23, #40. All datasets reporting bone onset were from spontaneous models of OA and all datasets reporting simultaneous onset were from the ACLT induced model of OA. The one dataset from the medial release model did not meet the criteria for best evidence synthesis and so is not included in this analysis. The results of the best evidence synthesis are summarized in Table 2.

**Table 2:** Summary of tissue onset of OA from best evidence synthesis

Model of OA	Onset of OA		
	Simultaneous	Bone	Cartilage
ACLT	5	⋯	⋯
Spontaneous	⋯	3	⋯
Medial release	⋯	⋯	⋯
Total	5	3	0

Abbreviations: ACLT, anterior cruciate ligament transection; OA, osteoarthritis.

### 3.5 Data analysis of the onset of OA pathology: Stratified by study model

#### 3.5.1 Anterior cruciate ligament transection

There were 16 datasets that used ACLT to induce OA and this included eight dog models and eight rabbit models. Seven datasets were of high quality and nine were of medium quality. There were no datasets of low quality. Nine datasets used multiple time points and seven used single time points. All 16 datasets used skeletally mature animals. Overall, 15 datasets reported simultaneous onset, one reported bone onset, and none reported cartilage onset. There were five datasets ranked as high quality that were in skeletally mature animals and used multiple time points. All of these datasets reported simultaneous onset. Additionally, since the criteria were all met in these five datasets, they were included in the best evidence synthesis analysis. In summary in the ACLT model, our best evidence synthesis indicates OA structural onset occurs simultaneously.

#### 3.5.2 Spontaneous

There were 19 datasets that used a spontaneous animal model to investigate OA and this included 15 guinea pig models, 3 horse models, and 1 cat model. Three datasets were of high quality, 14 were of medium quality, and 2 were of low quality. Twelve datasets used multiple time points and seven used single time points. Eleven datasets used skeletally mature animals, six used animals that reached skeletal maturity during data collection, and one used skeletally immature animals. There was also one dataset in which the skeletal maturity of the animals could not be determined. Overall, 15 datasets reported simultaneous onset, 4 reported bone onset, and none reported cartilage onset. There were three datasets ranked as high quality that were in skeletally mature animals and used multiple time points; since the criteria were all met in these three datasets, they were included in the best evidence synthesis analysis. These three datasets reported bone onset. In summary, in the spontaneous model, the best evidence synthesis suggests OA structural onset occurs in the bone.

#### 3.5.3 Groove model

There were four datasets that used the groove model to induce OA and this included three dog models and one sheep model. Two datasets were of high quality and two were of medium quality. There were no low‐quality datasets. Two datasets used multiple time points and two used single time points. All four datasets used skeletally mature animals. Overall, all four datasets reported simultaneous onset, none reported either bone or cartilage onset. There was one dataset ranked as high quality that was in skeletally mature animals and used multiple time points. However, because this model causes direct trauma to cartilage, datasets from this model were not included in the best evidence synthesis analysis. This dataset reported simultaneous onset.

#### 3.5.4 Meniscectomy

There were four datasets that used meniscectomy to induce OA and this included three rabbit models and one guinea pig model. Two datasets were of high quality and two were of medium quality. There were no datasets of low quality. Three datasets used multiple time points and one used a single time point. Three of the datasets used skeletally mature animals and one used skeletally immature animals. Overall, three datasets reported simultaneous onset, none reported bone onset, and one reported cartilage onset. No datasets that were high quality were in skeletally mature animals and had multiple time points, so no datasets from this group were included in the best evidence synthesis. There were two datasets that were ranked medium quality that were in skeletally mature animals and used multiple time points. These datasets both reported simultaneous onset.

#### 3.5.5 Overloading

There were three datasets that used overloading to induce OA and this included two rabbit models and one horse model. One dataset was of high quality, one was of medium quality, and one was of low quality. Two datasets had multiple time points and one used a single time point. All three datasets used skeletally mature animals. Overall, two datasets reported simultaneous onset and one reported bone onset. There was one dataset ranked as high quality that was in skeletally mature animals but used only one time point. This dataset reported simultaneous onset. However, because the high‐quality dataset had a singular time point, the datasets from this model are not included in the best evidence synthesis analysis.

#### 3.5.6 Subchondral fracture

One dataset used subchondral fracture to induce OA. It used the dog model, in skeletally mature animals, was ranked of medium quality, and used one time point. It reported a simultaneous onset. The dataset from this model was not included in the best evidence synthesis analysis.

#### 3.5.7 Medial release

One dataset used medial release as a method to induce OA. It used the dog model, used skeletally mature animals, was ranked of high quality, and only used one time point. It reported a simultaneous onset. The dataset from this model was not included in the best evidence synthesis analysis.

#### 3.5.8 Osteochondral fragment

One dataset used osteochondral fragments as a method to induce OA. It used the horse model, used skeletally mature animals, was ranked of high quality, and used multiple time points. It reported a simultaneous onset. However, because this model causes direct trauma to bone, the datasets from this model are not included in the best evidence synthesis analysis.

### 3.6 Analysis of the initiation of OA from studies with available meniscal data

We found four datasets that included meniscal pathology assessment in addition to bone and cartilage assessments. One induced model, a dog ACLT model Supporting Information C #30 reported bone onset. The onset of meniscal pathology was determined from MRI images by a radiologist describing the loss of the characteristic triangular shape and homogenous hypointense signal of the posterior part of the medial meniscus lost. The three spontaneous models of OA were in the Dunkin‐Hartley (DH) guinea pig animal model and all reported simultaneous onset. Sun et al^25^ reported the medial meniscal mineral density (ossification) and calcium content using female Strain 13 guinea pigs as a control for male DH guinea pigs. Tessier et al reported the MRI appearances of meniscus every 3 months for one year Supporting Information C #43 with no control animals. All three tissue pathologies began between 3 and 6 months. Thomsen et al described the meniscal ossification Supporting Information C #44. There were no control animals and the medial meniscal ossification was contrasted with variation from the lateral condyle at evaluated time points.

In summary, from these four datasets, OA structural onset appears to occur simultaneously in the cartilage, bone, and meniscus although available data to draw this conclusion are limited and questionable.

## 4 DISCUSSION

This is the first systematic literature review to explore the time sequence of tissue involvement in animal models of knee OA. We incorporated robust quality scoring and performed the best evidence synthesis. Overall, the high‐quality evidence base was small, thus any conclusions drawn are subject to some uncertainty and there is a need for further investigation by studies designed specifically for the purpose of uncovering the sequence of events in animal knee OA. Nonetheless, our findings suggest that the structural onset of OA occurs either simultaneously or in the subchondral bone. After the best evidence synthesis, all spontaneous models reported bone onset, while the induced models all reported simultaneous onset; none reported cartilage onset.

There is further evidence that supports bone onset and comes from studies including both spontaneous and induced animal models. The studies could not be included in this review because of exclusion criteria and/or also the presence of human knee data which was excluded in the original database search. In one such study, using the spontaneous DH guinea pig model, bone pathology was observed at 10 weeks^26^ before the onset of cartilage disease at 3 months (12 weeks),^27^ Supporting Information C #24. In dogs, using an ACLT/destabilization model, Boyd et al^28^ observed that trabecular bone morphometric pathology preceded cartilage pathology at 3 weeks. In the STR/ort mouse model of spontaneous OA and using three‐dimensional (3‐D) whole‐joint quantitative multimodal imaging, Stok et al concluded that it is too difficult to discern separate timelines for changes in bone and cartilage in early OA. However, they did demonstrate that there is a difference in bone morphology between the STR/ort mice and the CBA/1 mice which were/are used as a control species that do not develop OA. They go on to reason that these differences in bone morphology may be responsible for either the CBA/1 mice's resistance to OA or the STR/ort mice's vulnerability to OA.^29^ In humans, Davies‐Tuck et al^3^ reported amongst asymptomatic postmenopausal women tibial plateau bone enlargement occurs before significant pathological changes in cartilage.

Further evidence supporting simultaneous onset is presented in induced animal model studies that could not be included in this review because of the exclusion criteria. This includes an ACLT model in dogs by Widmer et al^30^ where both cartilage pathology and osteophytes were observed at 2 weeks. Also, in mice using the destabilization of the medial meniscus/destabilization model, Fang et al^31^ demonstrated that onset occurred at least simultaneously in bone and cartilage through demonstrating that subchondral bone remodeling was occurring at least as early as 2 weeks (earliest time point), while OARSI scores for cartilage were significantly different than sham‐operated mice at 2 weeks. Unfortunately, definitive human studies are lacking. There is a longitudinal study using serial knee MRI measurements in humans without symptoms of knee OA which reported that a higher baseline tibial bone area and osteophytes were associated with greater knee cartilage loss over 2 years.^32^, ^33^ However, at baseline between 13% and 28% of participants had a cartilage defect in the tibiofemoral compartment.^4^

No best evidence synthesis dataset reported cartilage onset in this systematic review. In all included datasets, only one supported cartilage onset of OA. Calvo et al used 2‐month‐old rabbits and studied them over the course of 10 weeks using the meniscectomy model of OA, harvesting at 6 and 10 weeks. Although this study was ranked as high quality, the animals were not skeletally mature which excluded it from being the best evidence synthesis dataset. Rabbits do not reach skeletal maturity until 8 or 9 months,^12^ resulting in an increased rate of tissue turnover and matrix remodeling in the bone. Although appropriate controls (sham‐operated and unoperated) were used to demonstrate increased cartilage thickness, while BMD and BV/BT stayed the same, the lack of change in the subchondral bone cannot be definitively attributed to cartilage onset of OA. Additionally, the time points for this dataset may not have been appropriate for the reported biphasic nature of bone changes. Batiste et al demonstrated that BMD decreased at 4 weeks and returned to normal levels by 8 weeks Supporting Information C #5 and Bouchgua et al corroborated this decrease in BMD at 4 weeks which then returned to normal by 10 weeks Supporting Information C #9, #10. Since the rabbits used in Calvo's study were skeletally immature, with increased rates of tissue turnover and matrix remodeling Supporting Information C #13, it is feasible that the bone could have changed and returned to normal by the first time point at 6 weeks.

Ultimately, since the meniscus has not been as robustly explored in animal models as other tissues, it is difficult to comment on the relationship between meniscus and the onset of OA. However, in addition to the lack of meniscal data reported, the use of meniscectomy as a method of inducing OA not only confounds evaluation of meniscal onset of OA but demonstrates that mechanical instability caused by the removal of the meniscus alone does induce OA.

Induced models of OA tended to report simultaneous OA onset in both cartilage and bone (regardless of whether best evidence synthesis was used or not) as highlighted in Figure 2. However, when best evidence synthesis was applied to spontaneous models, only bone onset was reported. This conclusion is supported by recent findings from Chen et al^34^ which reported marked changes in the subchondral bone between healthy control human knees and OA human knees and demonstrated that similar changes occurred in the guinea pig spontaneous model of OA.

There are important limitations to this study. Since the datasets included data from numerous animal models with a variety of induction methods, our analysis was performed cross‐species and cross‐induction methods. Differences within animal models include differing joint mechanics (eg, rabbits load knees mainly in the lateral compartment, whereas most other animals load knees more in the medial compartment), and different exercise regimes postsurgery (eg, rabbits and guinea pigs are allowed free cage movement, whereas generally dogs were allowed free pen movement with additional daily walks). Differences in induction methods include destabilization vs direct damage to the subchondral bone or cartilage.^35^

Another limitation relates to the variety of measurement techniques for both bone and cartilage and their reliability. For bone measurement techniques, measures of BMD and BV/BT were acquired in some cases by 2‐D histomorphometry and extrapolation by mathematical algorithms, while others were acquired from CT image data. For cartilage measuring techniques, over half of the datasets used the Mankin scoring criteria or a modification. However, the Mankin criteria have limitations in mild or moderate OA as it was developed on samples with advanced OA.^36^, ^37^, ^38^ Additionally, MRI was commonly used to assess cartilage degradation but may have variability of quantification related to issues, such as magnetic strength and sequence selection.^39^ The part of the joint examined sometimes varied: tibial plateau vs femoral head, medial vs lateral, and posterior vs anterior. In some cases, the cartilage and bone were not assessed from the same bones, both within a study and when comparing between studies. In cases where the same bone was assessed, sometimes the compartment used was different both within a study design and when comparing between studies. These differences could be significant because of loading characteristics within the joint differ between compartments, on different bones, and in different animal models.

For over a third of datasets (19/49), both cartilage and subchondral bone were analyzed at only one time point (usually the endpoint). This meant that many of the datasets were unable to assess longitudinal changes. This also highlights the limitation caused by the selection of time points in the datasets. Depending on when the data were acquired the changes in the cartilage or bone may have been missed, as mentioned previously.

The use of different controls comprises another limitation. Although the controls were previously validated or were validated within the study, many of the controls may not be optimal. In the case of spontaneous OA in guinea pigs, for example, both Bristol Strain 2 (BS2) and Weiser‐Maple (W‐M) guinea pigs (Strain 13) were used as nonsusceptible to OA controls for DH guinea pigs. BS2 are suitable controls for up to 36 weeks.^40^ Likewise, W‐M is viable controls for up to 12 months.^41^, ^42^ However, these are different breeds of guinea pigs and it is not unreasonable to question the strength of comparisons made between datasets that use different controls. Additionally, in the induced models of OA, there is controversy over which control is best to use. The three most common forms are contralateral unoperated joint, age‐matched healthy animal, or sham operation in additional animals. Nonoperated control animals are important negative controls but are often limited by both cost and may not be in line with the call to minimize numbers of animals in the guiding principles (the three Rs^43^) of humane animal research. Sham operations although acceptable can cause inflammation, pain, and in some cases, although gait because of the nature of a surgical procedure which opens the knee capsule. Contralateral unoperated controls although acceptable can lead to changed gait and loading patterns on the control joint as a result of the offloading of the injured limb.^12^

As stated previously, another limitation was some data were collected before epiphyseal closure, which confounds results concerning onset. There is a high potential that prior to epiphyseal closure the bone properties are changing. One example is that an increase in BV/BT has been shown prior to architectural adaptation in growing bone.^13^

Lastly, the sample sizes included in final analyses were typically small, both in the number of animals used and/or the number of samples assessed. Smaller sample sizes inherently increase the risk of bias.

In conclusion, this systematic review indicates that in animal studies conducted to date, the structural onset of knee OA occurs either simultaneously or in bone prior to cartilage pathology. In the best evidence, spontaneous knee OA model datasets reviewed, subchondral bone changes were reported at the onset of knee OA, while in the induced or posttraumatic models of knee OA, simultaneous changes in the subchondral bone were reported at the onset of knee OA. The limitations of available animal model data highlight the need for appropriate timing of joint tissue analysis and the analysis of menisci to gain a holistic assessment of knee OA pathogenesis. The observed patterns of tissue involvement in different knee OA phenotypes may inform future study design and interventions in humans.

## CONFLICT OF INTERESTS

The authors declare that there are no conflict of interests.

## AUTHOR CONTRIBUTIONS

MEC‐T: substantial contributions to design, acquisition of data, and analysis and interpretation of data; involved in drafting and revision of the manuscript; given approval of final version; and agreed to be accountable for all aspects of the work. AJB: substantial contributions to design, acquisition of data, and analysis and interpretation of data; involved in drafting and revision of the manuscript; given approval of final version; and agreed to be accountable for all aspects of the work. SW: substantial contributions to conception and design; involved in the revision of the manuscript; given approval of the final version; and agreed to be accountable for all aspects of the work. RKW: substantial contributions to conception and design; involved in the revision of the manuscript; given approval of the final version; and agreed to be accountable for all aspects of the work. PGC: substantial contributions to conception and design; involved in drafting and revision of the manuscript; given approval of the final version; and agreed to be accountable for all aspects of the work.

## Supporting information

Supporting information

Supporting information

Supporting information

Supporting information

Supporting information

## Data Availability

All data generated or analyzed during this study are included in this published article and its supplementary information files.

## References

[1] Guermazi A , Niu J , Hayashi D , et al. Prevalence of abnormalities in knees detected by MRI in adults without knee osteoarthritis: population based observational study (Framingham osteoarthritis study). BMJ. 2012;345:e5339. 22932918 10.1136/bmj.e5339 PMC3430365

[2] Goldring SR , Goldring MB . Changes in the osteochondral unit during osteoarthritis: structure, function and cartilage‐bone crosstalk. Nat Rev Rheumatol. 2016;12(11):632‐644. 27652499 10.1038/nrrheum.2016.148

[3] Davies‐tuck ML , Martel‐pelletier J , Wluka AE , et al. Meniscal tear and increased tibial plateau bone area in healthy post‐menopausal women. Osteoarthritis Cartilage. 2008;16(2):268‐271. 18093847 10.1016/j.joca.2007.10.018

[4] Ding C , Cicuttini F , Scott F , Cooley H , Jones G . Knee structural alteration and BMI: a cross‐sectional study. Obes Res. 2005;13(2):350‐361. 15800294 10.1038/oby.2005.47

[5] Englund M , Guermazi A , Gale D , et al. Incidental meniscal findings on knee MRI in middle‐aged and elderly persons. N Engl J Med. 2008;359(11):1108‐1115. 18784100 10.1056/NEJMoa0800777 PMC2897006

[6] Wang YY , Wluka AE , Pelletier JP , et al. Meniscal extrusion predicts increases in subchondral bone marrow lesions and bone cysts and expansion of subchondral bone in osteoarthritic knees. Rheumatology. 2010;49(5):997‐1004. 20181669 10.1093/rheumatology/keq034

[7] Ding CH , Martel‐Pelletier J , Pelletier JP , et al. Knee meniscal extrusion in a largely non‐osteoarthritic cohort: association with greater loss of cartilage volume. Arthritis Res Ther. 2007;9(2):R21. 17359552 10.1186/ar2132 PMC1906796

[8] Guermazi A , Eckstein F , Hayashi D , et al. Cartilage damage, bone marrow lesions and meniscal lesions predict quantitatively measured loss of cartilage over 30‐months: the most study. Osteoarthritis Cartilage. 2014;22:S356.

[9] Roemer FW , Kwoh CK , Hannon MJ , et al. What comes first? Multitissue Involvement leading to radiographic osteoarthritis: magnetic resonance imaging‐based trajectory analysis over four years in the osteoarthritis initiative. Arthritis Rheumatol. 2015;67(8):2085‐2096. 25940308 10.1002/art.39176 PMC4519416

[10] An YH , Freidman RJ . Animal Models in Orthopaedic Research. Boca Raton, FL: CRC Press; 1998.

[11] Gregory MH , Capito N , Kuroki K , Stoker AM , Cook JL , Sherman SL . A review of translational animal models for knee osteoarthritis. Arthritis. 2012;2012:1‐14. 10.1155/2012/764621 PMC3541554 23326663

[12] Teeple E , Jay GD , Elsaid KA , Fleming BC . Animal models of osteoarthritis: challenges of model selection and analysis. AAPS J. 2013;15(2):438‐446. 23329424 10.1208/s12248-013-9454-x PMC3675748

[13] Tanck E , Homminga J , VAN Lenthe GH , Huiskes R . Increase in bone volume fraction precedes architectural adaptation in growing bone. Bone. 2001;28(6):650‐654. 11425654 10.1016/s8756-3282(01)00464-1

[14] Libbin RM , Rivera ME . Regeneration of growth plate cartilage induced in the neonatal rat hindlimb by reamputation. J Orthop Res. 1989;7(5):674‐682. 2760739 10.1002/jor.1100070507

[15] Zomer HD , Trentin AG . Skin wound healing in humans and mice: challenges in translational research. J Dermatol Sci. 2018;90(1):3‐12. 29289417 10.1016/j.jdermsci.2017.12.009

[16] Seok J , Warren HS , Cuenca AG , et al. Genomic responses in mouse models poorly mimic human inflammatory diseases. Proc Natl Acad Sci U S A. 2013;110(9):3507‐3512. 23401516 10.1073/pnas.1222878110 PMC3587220

[17] Warren HS , Tompkins RG , Moldawer LL , et al. Mice are not men. Proc Natl Acad Sci U S A. 2015;112(4):E345. 25540422 10.1073/pnas.1414857111 PMC4313842

[18] Blaker CL , Clarke EC , Little CB . Using mouse models to investigate the pathophysiology, treatment, and prevention of post‐traumatic osteoarthritis. J Orthop Res. 2017;35(3):424‐439. 27312470 10.1002/jor.23343

[19] Huebner JL , Hanes MA , Beekman B , Tekoppele JM , Kraus VB . A comparative analysis of bone and cartilage metabolism in two strains of guinea‐pig with varying degrees of naturally occurring osteoarthritis. Osteoarthritis Cartilage. 2002;10(10):758‐767. 12359161 10.1053/joca.2002.0821

[20] de Vries RB , Wever KE , Avey MT , Stephens ML , Sena ES , Leenaars M . The usefulness of systematic reviews of animal experiments for the design of preclinical and clinical studies. ILAR J. 2014;55(3):427‐437. 25541545 10.1093/ilar/ilu043 PMC4276599

[21] Sena E , van der Worp HB , Howells D , Macleod M . How can we improve the pre‐clinical development of drugs for stroke? Trends Neurosci. 2007;30(9):433‐439. 17765332 10.1016/j.tins.2007.06.009

[22] Suokas A , Walsh D , Mcwilliams D , et al. Quantitative sensory testing in painful osteoarthritis: a systematic review and meta‐analysis. Osteoarthritis Cartilage. 2012;20(10):1075‐1085. 22796624 10.1016/j.joca.2012.06.009

[23] Festing MF , Altman DG . Guidelines for the design and statistical analysis of experiments using laboratory animals. ILAR J. 2002;43(4):244‐258. 12391400 10.1093/ilar.43.4.244

[24] Stroup DF , Berlin JA , Morton SC , et al. Meta‐analysis of observational studies in epidemiology: a proposal for reporting. JAMA. 2000;283(15):2008‐2012. 10789670 10.1001/jama.283.15.2008

[25] Sun Y , Scannell BP , Honeycutt PR , Mauerhan DR , Norton HJ , Hanley EN JR . Cartilage degeneration, subchondral mineral and meniscal mineral densities in Hartley and Strain 13 guinea pigs. Open Rheumatol J. 2015;9:65‐70. 26401159 10.2174/1874312901409010065 PMC4578142

[26] Zamli Z , ROBSON Brown K , Sharif M . Subchondral bone plate changes more rapidly than trabecular bone in osteoarthritis. Int J Mol Sci. 2016;17(9):1496. 10.3390/ijms17091496 PMC5037773 27618009

[27] Bendele AM , White SL , Hulman JF . Osteoarthrosis in guinea‐pigs—histopathologic and scanning electron‐microscopic features. Lab Anim Sci. 1989;39(2):115‐121. 2709799

[28] Boyd SK , Matyas JR , Wohl GR , Kantzas A , Zernicke RF . Early regional adaptation of periarticular bone mineral density after anterior cruciate ligament injury. J Appl Physiol. 2000;89(6):2359‐2364. 11090590 10.1152/jappl.2000.89.6.2359

[29] Stok KS , Pelled G , Zilberman Y , et al. Revealing the interplay of bone and cartilage in osteoarthritis through multimodal imaging of murine joints. Bone. 2009;45(3):414‐422. 19481620 10.1016/j.bone.2009.05.017

[30] Widmer WR , Buckwalter KA , Braunstein EM , Hill MA , Oconnor BL , Visco DM . Radiographic and magnetic‐resonance‐imaging of the stifle joint in experimental osteoarthritis of dogs. Veterinary Radiol Ultrasound. 1994;35(5):371‐384.

[31] Fang H , Huang L , Welch I . Early changes of articular cartilage and subchondral bone in the DMM mouse model of osteoarthritis. Sci Rep. 2018;8(1):2855. 29434267 10.1038/s41598-018-21184-5 PMC5809364

[32] Olsson O , Isacsson M , Engund M , Frobell R . (2014). Panorama of intra‐ and para‐articular injury after knee joint hemarthrosis e data from sub‐acute MR imaging findings in 1145 consecutive acute knee injuries. In Orthopedic Research Society Annual Meeting, New Orleans, LA.

[33] Driban JB , Eaton CB , Lo GH , Ward RJ , Lu B , Mcalindon TE . Association of knee injuries with accelerated knee osteoarthritis progression: data from the osteoarthritis initiative. Arthritis Care Res. 2014;66(11):1673‐1679. 10.1002/acr.22359 PMC4211979 24782446

[34] Chen Y , Hu Y , Yu YE , et al. Subchondral trabecular rod loss and plate thickening in the development of osteoarthritis. J Bone Miner Res. 2018;33(2):316‐327. 29044705 10.1002/jbmr.3313

[35] van der Kraan PM . Factors that influence outcome in experimental osteoarthritis. Osteoarthritis Cartilage. 2017;25(3):369‐375. 27616682 10.1016/j.joca.2016.09.005

[36] Ostergaard K , Andersen CB , Petersen J , Bendtzen K , Salter DM . Validity of histopathological grading of articular cartilage from osteoarthritic knee joints. Ann Rheum Dis. 1999;58(4):208‐213. 10364898 10.1136/ard.58.4.208 PMC1752860

[37] Ostergaard K , Petersen J , Andersen CB , Bendtzen K , Salter DM . Histologic/histochemical grading system for osteoarthritic articular cartilage: reproducibility and validity. Arthritis Rheum. 1997;40(10):1766‐1771. 9336409 10.1002/art.1780401007

[38] Rutgers M , VAN Pelt MJ , Dhert WJ , Creemers LB , Saris DB . Evaluation of histological scoring systems for tissue‐engineered, repaired and osteoarthritic cartilage. Osteoarthritis Cartilage. 2010;18(1):12‐23. 19747584 10.1016/j.joca.2009.08.009

[39] Palmer AJ , Brown CP , Mcnally EG , et al. Non‐invasive imaging of cartilage in early osteoarthritis. Bone Joint J. 2013;95‐B(6):738‐746. 10.1302/0301-620X.95B6.31414 23723266

[40] Anderson‐mackenzie JM , Quasnichka HL , Starr RL , Lewis EJ , Billingham ME , Bailey AJ . Fundamental subchondral bone changes in spontaneous knee osteoarthritis. Int J Biochem Cell Biol. 2005;37(1):224‐236. 15381164 10.1016/j.biocel.2004.06.016

[41] Huebner JL , Hanest MA , Beekman B , Tekoppele JM , Kraus VB . A comparative analysis of bone and cartilage metabolism in two strains of guinea‐pig with varying degrees of naturally occurring osteoarthritis. Osteoarthritis Cartilage. 2002;10(10):758‐767. 12359161 10.1053/joca.2002.0821

[42] Sato M , Wada M , Miyoshi N , et al. Hydroxyapatite maturity in the calcified cartilage and underlying subchondral bone of guinea pigs with spontaneous osteoarthritis: analysis by fourier transform infrared microspectroscopy. Acta Histochem Cytochem. 2004;37(2):101‐107.

[43] National Centre for the Replacement Refinement and Reduction of Animals in Research . The 3Rs [Online]. https://www.nc3rs.org.uk/the‐3rs. Accessed July 4, 2018.

