# Supplementary material for: Initiating factors for the onset of OA: A systematic review of animal bone and cartilage pathology in OA
Source: J Orthop Res. 2020 Feb 13;38(8):1810–8. doi: 10.1002/jor.24605 (PMC7383628; doi:10.1002/jor.24605)
Supplement: Supplementary file 2 — Supporting information [file JOR-38-1810-s002.docx]

**Appendix B. Checklist for Study Quality and Potential Risk of Bias. Each criterion received a score of 1 if it was reported satisfactorily and received a 0 if it was not. The maximum score was 11.**

1. Were animals included / groups similar at baseline in source (where appropriate), species, gender, age and weight?
2. Was care given to each group the same?
3. Was it a prospective study design?
4. Was (were) bone and cartilage evaluator(s) blinded to intervention?
5. Was (were) bone and cartilage evaluator(s) blinded to time point allocation?
6. Was an appropriate (validated) control (non-intervention) used to represent pre-intervention bone and cartilage tissue?
7. Were cartilage and bone measurements conducted on the same sample in >80% of samples from each group?
8. Standardized method was used for bone (not subjective assessment)?
9. Assessment was identical between samples of bone (i.e. one reader or ICC >0.81)? / Intra or inter reader reliability
10. Standardized method was used for cartilage (not subjective assessment)?
11. Assessment was identical between samples of cartilage (i.e. one reader or ICC >0.81)? / Intra or inter reader reliability
