# Supplementary material for: Initiating factors for the onset of OA: A systematic review of animal bone and cartilage pathology in OA
Source: J Orthop Res. 2020 Feb 13;38(8):1810–8. doi: 10.1002/jor.24605 (PMC7383628; doi:10.1002/jor.24605)
Supplement: Supplementary file 3 — Supporting information [file JOR-38-1810-s003.docx]

# References for Supplementary Table 1 (1-47)

1. Anastasiou, A., Skioldebrand, E., Ekman, S. and Hall, L.D. Ex vivo magnetic resonance imaging of the distal row of equine carpal bones: assessment of bone sclerosis and cartilage damage. *Vet Radiol Ultrasound.* 2003, **44**(5), pp.501-512.

2. Anderson-MacKenzie, J.M., Quasnichka, H.L., Starr, R.L., Lewis, E.J., Billingham, M.E.J. and Bailey, A.J. Fundamental subchondral bone changes in spontaneous knee osteoarthritis. *International Journal of Biochemistry and Cell Biology.* 2005, **37**(1), pp.224-236.

3. Anetzberger, H., Mayer, A., Glaser, C., Lorenz, S., Birkenmaier, C. and Müller-Gerbl, M. Meniscectomy leads to early changes in the mineralization distribution of subchondral bone plate. *Knee Surgery, Sports Traumatology, Arthroscopy.* 2014, **22**(1), pp.112-119.

4. Batiste, D.L., Kirkley, A., Laverty, S., Thain, L.M.F., Spouge, A.R., Gati, J.S., Foster, P.J. and Holdsworth, D.W. High-resolution MRI and micro-CT in an ex vivo rabbit anterior cruciate ligament transection model of osteoarthritis. *Osteoarthritis and Cartilage.* 2004, **12**(8), pp.614-626.

5. Batiste, D.L., Kirkley, A., Laverty, S., Thain, L.M.F., Spouge, A.R. and Holdsworth, D.W. Ex vivo characterization of articular cartilage and bone lesions in a rabbit ACL transection model of osteoarthritis using MRI and micro-CT. *Osteoarthritis and Cartilage.* 2004, **12**(12), pp.986-996.

6. Bohr, H. Experimental osteoarthritis in the rabbit knee joint. *Acta Orthopaedica.* 1976, **47**(5), pp.558-565.

7. Boileau, C., Martel-Pelletier, J., Abram, F., Raynauld, J.P., Troncy, É., D'Anjou, M.A., Moreau, M. and Pelletier, J.P. Magnetic resonance imaging can accurately assess the long-term progression of knee structural changes in experimental dog osteoarthritis. *Annals of the Rheumatic Diseases.* 2008, **67**(7), pp.926-932.

8. Borrelli Jr, J., Zaegel, M.A., Martinez, M.D. and Silva, M.J. Diminished cartilage creep properties and increased trabecular bone density following a single, sub-fracture impact of the rabbit femoral condyle. *Journal of Orthopaedic Research.* 2010, **28**(10), pp.1307-1314.

9. Bouchgua, M., Alexander, K., André d'Anjou, M., Girard, C.A., Carmel, E.N., Beauchamp, G., Richard, H. and Laverty, S. Use of routine clinical multimodality imaging in a rabbit model of osteoarthritis - part I. *Osteoarthritis and Cartilage.* 2009, **17**(2), pp.188-196.

10. Bouchgua, M., Alexander, K., Norman Carmel, E., d'Anjou, M.A., Beauchamp, G., Richard, H. and Laverty, S. Use of routine clinical multimodality imaging in a rabbit model of osteoarthritis - part II: bone mineral density assessment. *Osteoarthritis and Cartilage.* 2009, **17**(2), pp.197-204.

11. Brandt, K.D., Braunstein, E.M., Visco, D.M., O'Connor, B., Heck, D. and Albrecht, M. Anterior (cranial) cruciate ligament transection in the dog: a bona fide model of osteoarthritis, not merely of cartilage injury and repair. *J Rheumatol.* 1991, **18**(3), pp.436-446.

12. Brandt, K.D., Myers, S.L., Burr, D. and Albrecht, M. Osteoarthritic changes in canine articular cartilage, subchondral bone, and synovium fifty‐four months after transection of the anterior cruciate ligament. *Arthritis & Rheumatism.* 1991, **34**(12), pp.1560-1570.

13. Calvo, E., Palacios, I., Delgado, E., Ruiz-Cabello, J., Hernández, P., Sánchez-Pernaute, O., Egido, J. and Herrero-Beaumont, G. High-resolution MRI detects cartilage swelling at the early stages of experimental osteoarthritis. *Osteoarthritis and Cartilage.* 2001, **9**(5), pp.463-472.

14. Cantley, C.E.L., Firth, E.C., Delahunt, J.W., Pfeiffer, D.U. and Thompson, K.G. Naturally occurring osteoarthritis in the metacarpophalangeal joints of wild horses. *Equine Veterinary Journal.* 1999, **31**(1), pp.73-81.

15. D'Anjou, M.A., Moreau, M., Troncy, E., Martel-Pelletier, J., Abram, F., Raynauld, J.P. and Pelletier, J.P. Osteophytosis, subchondral bone sclerosis, joint effusion and soft tissue thickening in canine experimental stifle osteoarthritis: Comparison between 1.5 T magnetic resonance imaging and computed radiography. *Veterinary Surgery.* 2008, **37**(2), pp.166-177.

16. de Bri, E., Reinholt, F.P. and Svensson, O. Primary osteoarthrosis in guinea pigs: A stereological study. *Journal of Orthopaedic Research.* 1995, **13**(5), pp.769-776.

17. Dedrick, D.K., Goldstein, S.A., Brandt, K.D., O'Connor, B.L., Goulet, R.W. and Albrecht, M. A longitudinal study of subchondral plate and trabecular bone in cruciate‐deficient dogs with osteoarthritis followed up for 54 months. *Arthritis & Rheumatism.* 1993, **36**(10), pp.1460-1467.

18. Ding, M., Danielsen, C.C. and Hvid, I. Age-related three-dimensional microarchitectural adaptations of subchondral bone tissues in guinea pig primary osteoarthrosis. *Calcified Tissue International.* 2006, **78**(2), pp.113-122.

19. Florea, C., Malo, M.K.H., Rautiainen, J., Mäkelä, J.T.A., Fick, J.M., Nieminen, M.T., Jurvelin, J.S., Davidescu, A. and Korhonen, R.K. Alterations in subchondral bone plate, trabecular bone and articular cartilage properties of rabbit femoral condyles at 4 weeks after anterior cruciate ligament transection. *Osteoarthritis and Cartilage.* 2015, **23**(3), pp.414-422.

20. Huebner, J.L., Hanes, M.A., Beekman, B., TeKoppele, J.M. and Kraus, V.B. A comparative analysis of bone and cartilage metabolism in two strains of guinea-pig with varying degrees of naturally occurring osteoarthritis. *Osteoarthritis Cartilage.* 2002, **10**(10), pp.758-767.

21. Intema, F., Hazewinkel, H.A.W., Gouwens, D., Bijlsma, J.W.J., Weinans, H., Lafeber, F.P.J.G. and Mastbergen, S.C. In early OA, thinning of the subchondral plate is directly related to cartilage damage: Results from a canine ACLT-meniscectomy model. *Osteoarthritis and Cartilage.* 2010, **18**(5), pp.691-698.

22. Intema, F., Sniekers, Y.H., Weinans, H., Vianen, M.E., Yocum, S.A., Zuurmond, A.M.M., DeGroot, J., Lafeber, F.P. and Mastbergen, S.C. Similarities and discrepancies in subchondral bone structure in two differently induced canine models of osteoarthritis. *Journal of Bone and Mineral Research.* 2010, **25**(7), pp.1650-1657.

23. Jia, L., Chen, J., Wang, Y., Liu, Y., Zhang, Y. and Chen, W. Magnetic resonance imaging of osteophytic, chondral, and subchondral structures in a surgically-induced osteoarthritis rabbit model. *PLoS One.* 2014, **9**(12), p.e113707.

24. Jimenez, P.A., Glasson, S.S., Trubetskoy, O.V. and Haimes, H.B. Spontaneous osteoarthritis in Dunkin Hartley guinea pigs: Histologic, radiologic, and biochemical changes. *Laboratory Animal Science.* 1997, **47**(6), pp.598-601.

25. Kuroki, K., Cook, C.R. and Cook, J.L. Subchondral bone changes in three different canine models of osteoarthritis. *Osteoarthritis and Cartilage.* 2011, **19**(9), pp.1142-1149.

26. Lacourt, M., Gao, C., Li, A., Girard, C., Beauchamp, G., Henderson, J.E. and Laverty, S. Relationship between cartilage and subchondral bone lesions in repetitive impact trauma-induced equine osteoarthritis. *Osteoarthritis and Cartilage.* 2012, **20**(6), pp.572-583.

27. Lahm, A., Kreuz, P.C., Oberst, M., Maier, D., Haberstroh, J. and Uhl, M. Subchondral and trabecular bone remodeling in canine experimental osteoarthritis. *Archives of Orthopaedic and Trauma Surgery.* 2006, **126**(9), pp.582-587.

28. Lee, J.H., Dyke, J.P., Ballon, D., Ciombor, D.M., Rosenwasser, M.P. and Aaron, R.K. Subchondral fluid dynamics in a model of osteoarthritis: use of dynamic contrast-enhanced magnetic resonance imaging. *Osteoarthritis and Cartilage.* 2009, **17**(10), pp.1350-1355.

29. Leijon, A., Ley, C.J., Corin, A. and Ley, C. Cartilage lesions in feline stifle joints - Associations with articular mineralizations and implications for osteoarthritis. *Res Vet Sci.* 2017, **114**, pp.186-193.

30. Libicher, M., Ivancic, M., Hoffmann, V. and Wenz, W. Early changes in experimental osteoarthritis using the Pond-Nuki dog model: Technical procedure and initial results of in vivo MR imaging. *European Radiology.* 2005, **15**(2), pp.390-394.

31. Mastbergen, S.C., Pollmeier, M., Fischer, L., Vianen, M.E. and Lafeber, F.P.J.G. The groove model of osteoarthritis applied to the ovine fetlock joint. *Osteoarthritis and Cartilage.* 2008, **16**(8), pp.919-928.

32. Messner, K., Fahlgren, A., Ross, I. and Andersson, B. Simultaneous changes in bone mineral density and articular cartilage in a rabbit meniscectomy model of knee osteoarthrosis. *Osteoarthritis and Cartilage.* 2000, **8**(3), pp.197-206.

33. Muraoka, T., Hagino, H., Okano, T., Enokida, M. and Teshima, R. Role of subchondral bone in osteoarthritis development: A comparative study of two strains of guinea pigs with and without spontaneously occurring osteoarthritis. *Arthritis and Rheumatism.* 2007, **56**(10), pp.3366-3374.

34. Pastoureau, P., Leduc, S., Chomel, A. and De Ceuninck, F. Quantitative assessment of articular cartilage and subchondral bone histology in the meniscectomized guinea pig model of osteoarthritis. *Osteoarthritis and Cartilage.* 2003, **11**(6), pp.412-423.

35. Pinilla, M.J., Tranquille, C.A., Blunden, A.S., Chang, Y.M., Parkin, T.D.H. and Murray, R.C. Histological Features of the Distal Third Metacarpal Bone in Thoroughbred Racehorses, With and Without Lateral Condylar Fractures. *J Comp Pathol.* 2017, **157**(1), pp.1-10.

36. Radin, E.L., Parker, H.G., Pugh, J.W., Steinberg, R.S., Paul, I.L. and Rose, R.M. Response of joints to impact loading. 3. Relationship between trabecular microfractures and cartilage degeneration. *J Biomech.* 1973, **6**(1), pp.51-57.

37. Sato, M., Wada, M., Miyoshi, N., Imamura, Y., Noriki, S., Uchida, K., Kobayashi, S., Yayama, T., Negoro, K., Fujimoto, M., Fukuda, M. and Baba, H. Hydroxyapatite maturity in the calcified cartilage and underlying subchondral bone of guinea pigs with spontaneous osteoarthritis: Analysis by Fourier transform infrared microspectroscopy. *Acta Histochemica Et Cytochemica.* 2004, **37**(2), pp.101-107.

38. Serink, R.T., Nachemson, A. and Hansson, G. the effect of impact loading on rabbit knee joints. *Acta Orthopaedica.* 1977, **48**(3), pp.250-262.

39. Smith, A.D., Morton, A.J., Winter, M.D., Colahan, P.T., Ghivizzani, S., Brown, M.P., Hernandez, J.A. and Nickerson, D.M. MAGNETIC RESONANCE IMAGING SCORING OF AN EXPERIMENTAL MODEL OF POST-TRAUMATIC OSTEOARTHRITIS IN THE EQUINE CARPUS. *Vet Radiol Ultrasound.* 2016, **57**(5), pp.502-514.

40. Sniekers, Y.H., Intema, F., Lafeber, F.P.J.G., Van Osch, G.J.V.M., Van Leeuwen, J.P.T.M., Weinans, H. and Mastbergen, S.C. A role for subchondral bone changes in the process of osteoarthritis; A micro-CT study of two canine models. *BMC Musculoskeletal Disorders.* 2008, **9**.

41. Stok, K.S., Besler, B.A., Steiner, T.H., Escudero, A.V.V., Zulliger, M.A., Wilke, M., Atal, K., Quintin, A., Koller, B., Müller, R. and Nesic, D. Three-dimensional quantitative morphometric analysis (QMA) for in situ joint and tissue assessment of osteoarthritis in a preclinical rabbit disease model. *PLoS One.* 2016, **11**(1).

42. Sun, Y., Scannell, B.P., Honeycutt, P.R., Mauerhan, D.R., Norton, H.J. and Hanley, E.N., Jr. Cartilage degeneration, subchondral mineral and Meniscal mineral densities in Hartley and strain 13 Guinea pigs. *Open Rheumatology Journal.* 2015, **9**, pp.65-70.

43. Tessier, J.J., Bowyer, J., Brownrigg, N.J., Peers, I.S., Westwood, F.R., Waterton, J.C. and Maciewicz, R.A. Characterisation of the guinea pig model of osteoarthritis by in vivo three-dimensional magnetic resonance imaging. *Osteoarthritis Cartilage.* 2003, **11**(12), pp.845-853.

44. Thomsen, J.S., Straarup, T.S., Danielsen, C.C., Oxlund, H. and Brüel, A. Relationship between articular cartilage damage and subchondral bone properties and meniscal ossification in the Dunkin Hartley guinea pig model of osteoarthritis. *Scandinavian Journal of Rheumatology.* 2011, **40**(5), pp.391-399.

45. Wang, T., Wen, C.Y., Yan, C.H., Lu, W.W. and Chiu, K.Y. Spatial and temporal changes of subchondral bone proceed to microscopic articular cartilage degeneration in guinea pigs with spontaneous osteoarthritis. *Osteoarthritis and Cartilage.* 2013, **21**(4), pp.574-581.

46. Yan, J.Y., Zhang, Y.Z., Tian, F.M., Wang, W.Y., Cheng, Y., Xu, H.F., Song, H.P. and Zhang, L. Age dependent changes in cartilage matrix, subchondral bone mass, and estradiol levels in blood serum, in naturally occurring osteoarthritis in guinea pigs. *International Journal of Molecular Sciences.* 2014, **15**(8), pp.13578-13595.

47. Zhao, W., Wang, T., Luo, Q., Chen, Y., Leung, V.Y.L., Wen, C., Shah, M.F., Pan, H., Chiu, K., Cao, X. and Lu, W.W. Cartilage degeneration and excessive subchondral bone formation in spontaneous osteoarthritis involves altered TGF-β signaling. *Journal of Orthopaedic Research.* 2016, **34**(5), pp.763-770.
